# Comprehensive Characterization of B-Box Zinc Finger Genes in *Citrullus lanatus* and Their Response to Hormone and Abiotic Stresses

**DOI:** 10.3390/plants12142634

**Published:** 2023-07-13

**Authors:** Xinsheng Wang, Huidan Guo, Zhi Jin, Yina Ding, Meng Guo

**Affiliations:** 1 School of Enology and Horticulture, Ningxia University, Yinchuan 750021, China; wxs19980@163.com (X.W.); 14709536107@163.com (Z.J.); 15349670751@163.com (Y.D.); 2 College of Horticulture and Landscape, Henan Institute of Science and Technology, Xinxiang 453003, China; guohd2021@126.com; 3 Key Laboratory of Modern Molecular Breeding for Dominant and Special Crops in Ningxia, Yinchuan 750021, China; 4 Ningxia Modern Facility Horticulture Engineering Technology Research Center, Yinchuan 750021, China; 5 Ningxia Facility Horticulture Technology Innovation Center, Ningxia University, Yinchuan 750021, China

**Keywords:** BBX, watermelon, gene family, expression profile, abiotic stress

## Abstract

Plant B-BOX (BBX) zinc finger transcription factors play crucial roles in growth and development and the stress response. Although the BBX family has been characterized in various plants, systematic analysis in watermelon is still lacking. In this study, 25 watermelon *ClBBX* genes were identified. ClBBXs were grouped into five clades (Clade I, II, III, IV, and V) based on their conserved domains and phylogenetic relationships. Most of the ClBBXs (84%) might be localized in the nuclei or cytoplasm. The classification of ClBBXs was consistent with their gene structures. They were unevenly distributed in nine chromosomes except for Chr4 and Chr10, with the largest number of six members in Chr2. Segmental duplications were the major factor in *ClBBX* family expansion. Some *BBXs* of watermelon and *Arabidopsis* evolved from a common ancestor. In total, 254 hormonal and stress-responsive *cis* elements were discovered in *ClBBX* promoters. *ClBBXs* were differentially expressed in tissues, and the expression levels of *ClBBX15* and *16* were higher in aboveground tissues than in roots, while the patterns of *ClBBX21a*, *21b*, *21c*, *28* and *30b* were the opposite. With salicylic acid, methyl jasmonate and salt stress conditions, 17, 18 and 18 *ClBBXs* exhibited significant expression changes, respectively. In addition, many *ClBBXs*, including *ClBBX29b*, *30a* and *30b*, were also responsive to cold and osmotic stress. In summary, the simultaneous response of multiple *ClBBXs* to hormonal or abiotic stress suggests that they may have functional interactions in the stress hormone network. Clarifying the roles of key ClBBXs in transcriptional regulation and mediating protein interactions will be an important task. Our comprehensive characterization of the watermelon ClBBX family provides vital clues for the in-depth analysis of their biological functions in stress and hormone signaling pathways.

## 1. Introduction

Plant transcription factors (TFs) function mainly through transcriptional regulation [1,2]. With the completion of plant genome sequencing, more and more TF families have been identified. As one of the largest TF families, the plant zinc finger TF family is made of crucial regulators of growth and development and the stress response [3]. The zinc finger proteins harbor a highly conserved zinc finger domain that requires the coordination of zinc ions to stabilize its structure [4]. Zinc finger TFs can be classified into several families according to the number and location of cysteine (Cys) and histidine (His) residues that bind the zinc ions [4,5]. Among them, the functional diversity of the B-box (BBX) family has attracted much attention. BBXs contain one or two conserved B-box domains (B-box1 and B-box2) of approximately 40 residues in length in the N-terminal, and the B-box domains in plants are thought to be crucial in mediating transcription activity and protein interactions [3]. The differences between two B-box motifs may be attributed to segmental duplications and internal deletion events [6]. In some cases, a CCT (CONSTANS, CO-like and TOC1) domain is present in the C terminus of BBXs. The highly conserved CCT domain functions in transcriptional regulation and nuclear protein transport [6]. *Arabidopsis* and grapevine BBXs were grouped into five structure groups (I–V) based on the number of B-box and CCT domains, and significantly clustered with dicotyledonous plants rather than rice, as is consistent with their position in plant classification [7,8].

BBX proteins widely participate in physiological and biochemical processes in plants [3,9,10]. For instance, BBXs regulated the light signaling pathways by modulating the activity of ELONGATED HYPOCOTYL 5 (HY5) and CONSTITUTIVELY PHOTOMORPHOGENIC 1 (COP1) [11]. In addition, many *Arabidopsis* BBX members functioned in a HY5-dependent manner [12]. They formed a transcriptional feedback loop with HY5, regulating photomorphogenic development [13]. Both pear PpBBX16 and apple MdBBX37 regulated anthocyanin synthesis through synergistic action with MYBs and HY5 [14,15]. Additionally, MdBBX37 also enhanced jasmonic acid (JA)-mediated cold resistance [16]. Sweet cherry PavBBX6 and PavBBX9 positively regulated anthocyanin and abscisic acid (ABA) accumulation [17]. Chrysanthemum *CmBBX13* delayed flowering time [18], while *CmBBX22* negatively regulated drought resistance [19]. Ginkgo *GbBBX25* enhanced the salt adaptability of transgenic poplar [20]. These reports confirm the critical role of the plant BBX family in the regulatory network that controls growth and development [3].

The identification of *BBX* family members is the prerequisite for a comprehensive analysis of the interaction among different BBXs. The characterization of the *BBX* family in many plants has been reported. The number of cotton *BBX*s ranged from 17 (*Gossypium arboreum*) to 37 (*G. hirsutum*), and the expansion of this family in cotton occurred mainly through segmental replication [21]. The 25 *VviBBXs* in grapevine might participate in powdery mildew infestation, hormone signaling, and seed abortion [8]. Most of the 29 tomato *SlBBXs* were induced by drought or heat stress, and some were strongly induced by ABA, gibberellic acid (GA), or ethephon [22]. The potato *StBBX* family (consisting of 30 members) might be made up of the key players in the circadian rhythm network [23]. Briefly, 12 of the 25 pear *PbBBXs* were specifically expressed in pollen tubes [24]. Most of the 23 pepper *CaBBXs* might regulate anthocyanin accumulation [25]. However, to our knowledge, there has been no comprehensive analysis of watermelon *BBX* (*ClBBX*) family to date.

Watermelon (*Citrullus lanatus*) is a cucurbitacea crop and one of the top five most commonly consumed fresh fruits [26]. It contains glucose, fructose, ascorbic acid, protein, inorganic salt and so on, having a high nutritional value and therapeutic value. TFs including the BBX family may be widely involved in biological processes in watermelon, but studies on the function of ClBBXs are relatively lacking. The publication of watermelon genome facilitates the identification of the *ClBBX* family. In this study, 25 genes encoding watermelon ClBBXs were identified. The gene structure, domains, phylogeny, and synteny were determined. The *cis* elements involved in stress and hormone responsive were analyzed in *ClBBX* promoters. In addition, the expression of *ClBBXs* in tissues and their response to salicylic acid (SA), methyl jasmonate (MeJA) and abiotic stresses (salt, cold, and osmotic stress) were analyzed. Our work will be helpful for the further functional analysis of watermelon ClBBXs.

## 2. Results

### 2.1. Identification of Watermelon BBXs

To identify watermelon *BBXs*, the HMM profile of the B-box zinc finger domain (Pfam: PF00643) was used as a BLAST query against the Cucurbit Genomics Database (CuGenDB), and the *Arabidopsis* BBXs were also used as a BLAST query against CuGenDB. Briefly, 31 candidate *BBX* sequences were obtained, of which 6 sequences were removed due to their e-value of >0.01. The remaining protein sequences were assessed for the presence of B-box zinc finger domains through Pfam and SMART. Finally, 25 watermelon *BBX* candidate members were identified and designated as *ClBBXs*. The structure type of ClBBXs (Structure I, II, III, IV, and V groups) was based on the classification rules of BBX family members in *Arabidopsis* (Table 1). The coding sequence (CDS) sizes for *ClBBXs* ranged from 378 (*ClBBX29b*) to 1,503 bp (*ClBBX27*), the length of deduced proteins ranged from 125 to 500 amino acids, and the molecular weights ranged from 13.42 to 56.18 kDa. The number of introns in *ClBBXs* ranged from 0 to 4. The isoelectric points of ClBBXs ranged from 4.38 (ClBBX29a) to 7.49 (ClBBX15), and only three proteins (ClBBX29b, 30a, and 30b) belonging to Structure V were stable. Additionally, most ClBBXs were predicted to be localized in the nucleus or cytoplasm, while ClBBX30b might function primarily in chloroplasts (Table 1).

### 2.2. Conserved Domains and Phylogenetic Analysis of ClBBX Family

**Figure 1:**
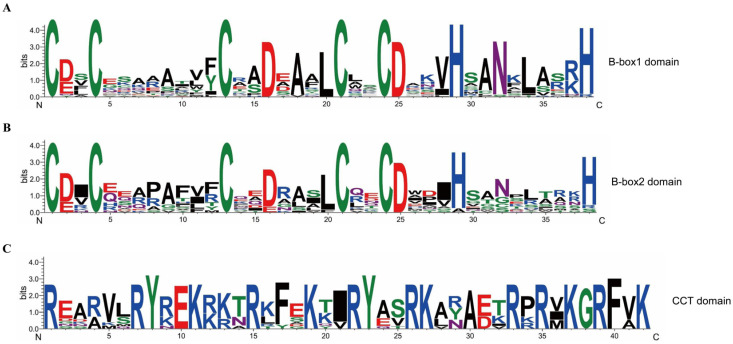
Sequence conservation within the conserved domains of watermelon ClBBXs. The sequence alignment of B-box1, B-box2 and CCT domains are shown in (**A**–**C**), respectively. The *x*-axis represents the positions, and the height of letters in the *y*-axis indicates the degree of conservation of each residue across ClBBXs.

Among the 25 ClBBXs, 4 members each belonged to Structure I and II groups, 3 members belonged to Structure III groups, 8 members belonged to Structure IV group, and 6 members belonged to Structure V group. Except for ClBBX3 from the Structure I group which had no B-box2 domain, all other ClBBXs from Structure I and II groups contained B-box1, B-box2, and CCT domains. All members of the Structure III group contained B-box1 and CCT domains, those of the Structure IV group contained two B-box domains, while those of the Structure V group had only B-box1 (Table 1, Figure S1B). As shown in Figure 1, the conserved sequences of B-box1 and B-box2 of watermelon ClBBXs were **C**-X_2_-**C**-X_7–8_-**C**-X_2_-**D**-X-**A**-X-**L**-**C**-X_2_-**C**-**D**-X_3_-**H**-X_2_-**N**-X_5_-**H** and **C**-X_2_-**C**-X_8_-**C**-X_2_-**D**-X_3_-**L**-**C**-X_2_-**C**-**D**-X_3_-**H**-X_6–8_-**H**, respectively. Additionally, the CCT domain of 11 ClBBXs with the form of **R**-X_5_-**R**-**Y**-X-**E**-**K**-X_3_-**R**-X-**F**-X-**K**-X_2_-**R**-**Y**-X_2_-**R**-**K**-X_2_-**A**-X_2_-**R**-X-**R**-X-**K**-**G**-**R**-**F**-X-**K** was also conserved. B-box1 and B-box2 shared similar conserved domains, and cysteine residues (C) in zinc fingers and arginine residues (R) in CCT domains were highly conserved.

**Figure 2:**
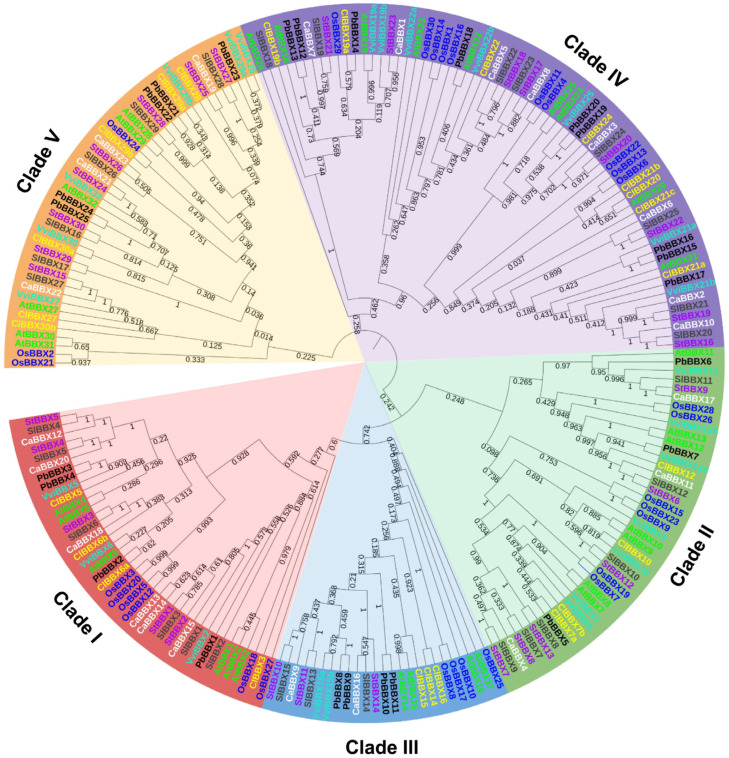
Phylogenetic tree of watermelon, *Arabidopsis*, tomato, pepper, potato, grapevine, pear, and rice BBXs. The sequences of BBXs were used for the construction of the unrooted neighbor-joining (NJ) phylogenetic tree using the MEGA-X program with pairwise deletion and Poisson correction. The member of watermelon (prefixed by Cl), *Arabidopsis* (At), tomato (Sl), pepper (Ca), potato (St), grapevine (Vvi), pear (Pb), and rice (Os) BBX family were used, and the five clades (Clade I–V) are marked by different colors.

To discover the evolutionary relationships and divergence of ClBBX members, 219 BBXs in total, including 32 from *Arabidopsis*, 25 from grapevine, 25 from pear, 23 from pepper, 30 from potato, 30 from rice and 29 from tomato, were used to construct a phylogenetic tree (Figure 2). BBXs were grouped into five distinct clades (Clade I–V), including 40 members in Clade I, 43 in II, 25 in III, 67 in IV and 44 in V, which corresponded to the structural groups of ClBBXs (Table 1, Figure S1A). In each clade, BBXs from Solanaceae (tomato, pepper, and potato) were clustered together significantly. Compared with rice, most of watermelon ClBBXs were closer to BBX proteins of dicotyledon, which was consistent with the botanical classification. Although watermelon ClBBX19a and pear PbBBX14 were clustered together, rice OsBBX29 was closer to both than were other Clade IV members. Moreover, an exception was also found in Clade V. Watermelon ClBBX29b was clustered with rice OsBBX24, but not with other dicotyledonous homologous proteins (Figure 2).

### 2.3. Gene Structure, Chromosomal Localization and Gene Duplications of ClBBXs 

Briefly, 5 (20%) of the 25 *ClBBXs* had no introns, 9 members (36%) had one intron, 11 genes (44%) had two or more introns, and only *ClBBX27* from Clade V had four introns (Table 1, Figure S1C). All four members from Clade I (*ClBBX3*, *5*, *6a*, and *6b*) and three members from Clade III (*ClBBX14*, *15*, and *16*) had only one intron, while all four genes of Clade II (*ClBBX7a*, *7b*, *10*, and *12*) contained three introns. The genes without introns belonged mainly to Clade V (*ClBBX28*, *29b*, *30a*, and *30b*) and only *ClBBX21b* belonged to Clade IV. The structures of Clade IV genes were relatively complex, with seven out of eight genes containing at least one intron. The structures of *ClBBXs* may be related to their phylogenetic classification.

Watermelon *ClBBXs* were widely distributed in 9 out of 11 chromosomes except for Chr4 and Chr10 (Table 1, Figure S2). Chr2 contained the most *ClBBXs* (6), while Chr3 and Chr9 both possessed only one gene. Two *ClBBXs* were distributed on Chr1 and Chr11, respectively. Chr5, Chr6, and Chr8 each contained three genes, and four members were distributed on Chr7.

**Figure 3:**
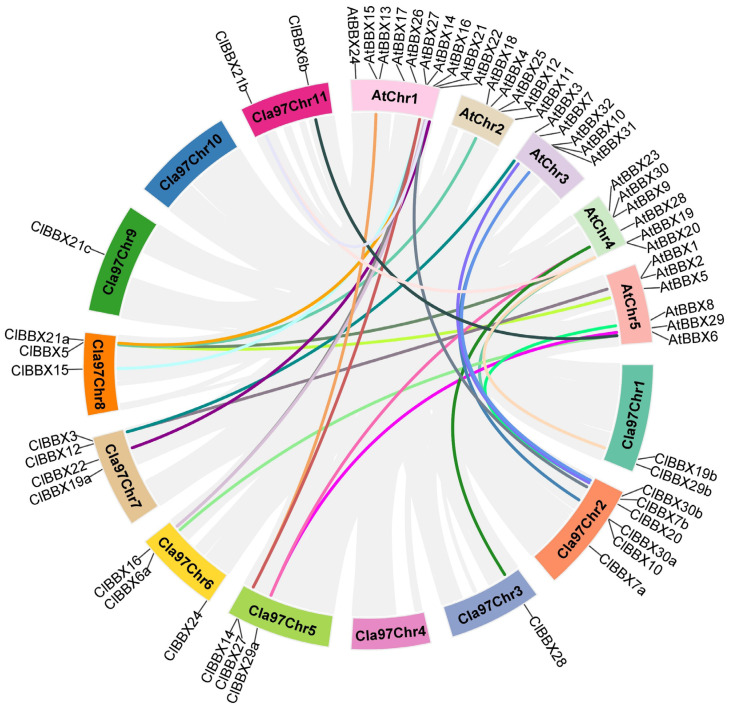
Synteny analysis of *BBXs* between watermelon and *Arabidopsis*. Syntenic occurrences of *BBXs* (*ClBBXs* and *AtBBXs*) are represented by colored lines.

Gene duplication events contribute to genome evolution; therefore, the duplications of *ClBBXs* was analyzed. Nine segmental duplication events were detected, but no tandem duplication events were identified (Figure S2 and Table S1), suggesting that segmental duplication events contribute to *ClBBX* family evolution. Additionally, 27 gene pairs were found between watermelon and *Arabidopsis*, comprising 18 *ClBBXs* and 18 *AtBBXs* (Figure 3, Table S2). Among them, nine orthologous pairs were found, and nine orthologous gene pairs with one *ClBBX* gene corresponding to multiple *AtBBXs* were identified. Furthermore, six orthologous gene pairs with one *AtBBX* gene corresponding to multiple *ClBBXs* were found. These results suggest that more than half of the *BBXs* appeared prior to differentiation between watermelon and *Arabidopsis*. The ratios of Ka/Ks of all segmentally duplicated gene pairs were less than 1.0 (Tables S4 and S5), indicating that the pairs had evolved primarily under purifying selection. The duplication events occurred between 84.9 and 204.3 million years ago (Mya) in watermelon (Table S1), and between 152.5 and 256.1 Mya in watermelon and *Arabidopsis* (Table S2).

### 2.4. Analysis of cis Elements in ClBBX Promoters and GO Enrichment Analysis of ClBBXs

**Figure 4:**
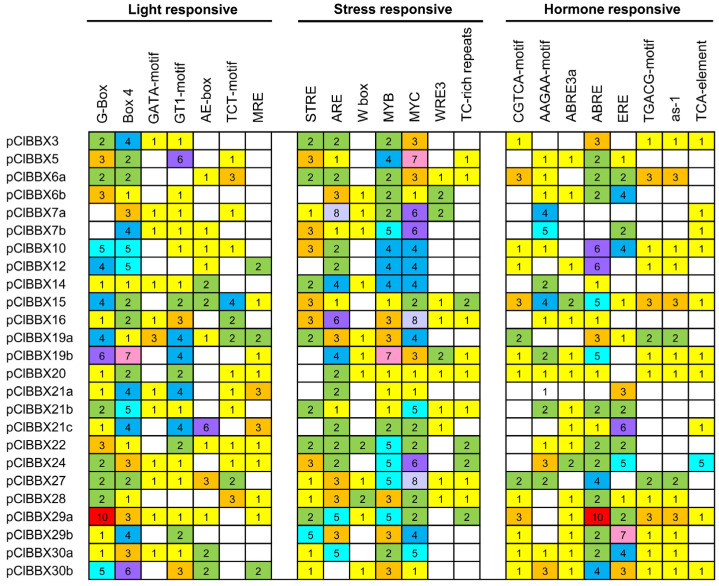
Predicted *cis* elements in *ClBBX* promoters. The different number of *cis* elements is marked by different colors.

To investigate the potential function of watermelon *ClBBXs*, the *cis*-acting elements in all *ClBBX* promoters were analyzed via PlantCARE. Except for the basic *cis* elements CAAT-box and TATA-box, 22 other *cis* elements were identified in more than 10 *ClBBX* promoter regions. These *cis* elements consisted of light-, stress- and hormone-responsive elements (Figure 4, Table 2). Among the seven light-responsive elements, only Box 4 was distributed in promoters of all *ClBBXs* and the highest number was found in *ClBBX19b* (7). G-box elements were not found in *ClBBX7a* and *7b* promoters, while the GT1-motif was absent in the promoters of *ClBBX6a*, *12* and *28*. The largest number of G-box and GT1-motifs existed in the promoter of *ClBBX29a* (10) and *ClBBX21c* (6), respectively (Figure 4). Four other light-responsive elements, including GATA-motif, AE-box, TCT-motif and MRE, were identified in 12, 13, 14 and 12 gene promoters, respectively (Table 2). Among the seven stress responsive elements, both MYB and MYC elements existed in all *ClBBX* promoters, and ARE was identified in all members except for the *ClBBX30b* promoter. The number of STRE elements in the *ClBBX29b* promoter (5) was the highest among that of the 19 *ClBBX* promoters, while the number of W-box, WRE3 and TC-rich repeat elements in each promoter was less than three (Figure 4). Three (AAGAA-motif, ABRE3a and ABRE), two (CGTCA-motif and TGACG-motif), two (as-1 and TCA-element) and one (ERE) of the eight hormone-responsive elements were ABA, MeJA, SA and ethylene-responsive elements, respectively (Table 2). Compared with other gene promoters, the number of MeJA response elements, the CGTCA-motif (3), TGACG-motif (3) and SA response element as-1 (3) in the promoters of three genes (*ClBBX6a*, *15* and *29a*) was the highest. The largest number of AAGAA-motif, ABRE, ERE and TCA-elements was found in promoters of *ClBBX7b* (5), *ClBBX29a* (10), *ClBBX29b* (7) and *ClBBX24* (5), respectively. Furthermore, among the 16 *ClBBX* promoters containing ABRE3a elements, only *ClBBX15* and *ClBBX24* promoters contained two elements, and the other 14 promoters contained only one (Figure 4). 

The enrichment analysis of ClBBXs was performed (Figure S3) and conducted on the assigned GO terms with the corrected *p*-value of <0.05 [26]. In terms of molecular function, all ClBBXs were assigned in binding. In the cellular component, intracellular and cellular parts were highly enriched in ClBBX members. Among them, 12 members (48%) were also assigned to the nucleus. Twenty-three ClBBX members (92%) were assigned to the biological process.

### 2.5. Expression Analysis of ClBBXs in Different Tissues

**Figure 5:**
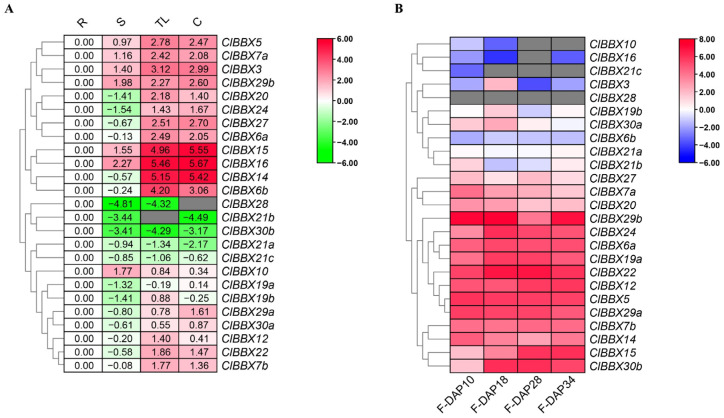
Expression analysis of *ClBBXs* in different tissues by qRT-PCR (**A**) and RNA-Seq (**B**). In (**A**), R, roots; S, stems; TL, true leaves; C, cotyledons; qRT-PCR data in were normalized using watermelon *yellow-leaf-specific proein8* (*ClYLS8*) and *β-actin* (*ClACT*), and were shown relative to those for roots; the relative expression levels were calculated using the −ΔΔCT method; the gray blocks indicate that the values were not detected. In (**B**), raw data were from FPKM values of watermelon fruits (cultivar Dumara); the FPKM values for zero were indicated in gray, and the other data were normalized using log2. DAP10, DAP18, DAP28, and DAP34 represented the fruits on the 10th, 18th, 28th and 34th day after pollination, respectively; F, fruits. The heat map was created using TBtools. A cluster dendrogram is marked on the left.

To investigate the potential functions of *ClBBXs* during watermelon development, qRT-PCR was used to determine the global transcription levels of 25 *ClBBXs* in roots, stems, true leaves, and cotyledons (Figure 5A). Among them, the relative expression levels of seven *ClBBXs* (*ClBBX3*, *5*, *7a*, *10*, *15*, *16*, and *29b*) (28%) were higher in shoots than in roots. Except for *ClBBX10* (the only gene with the highest transcription level in stems), the levels of six other genes in stems were lower than those in cotyledons and true leaves. Conversely, five genes (*ClBBX21a*, *21b*, *21c*, *28*, and *30b*) (20%) were preferentially expressed in roots rather than in shoots. The genes preferentially expressed in cotyledons (9) and true leaves (10) accounted for 76% of the total 25 genes. It was noteworthy that the transcription levels of *ClBBX14*, *ClBBX15* and *ClBBX16* in cotyledons were the highest (40~50-fold that in roots) and their levels in true leaves were much higher than those in roots (30~44-fold).

To analyze the potential function of *ClBBXs* in fruit development, an expression atlas was created for the genes against the RNA-seq data of four developmental stages of watermelon fruits, including white fruits (10 days after pollination, F-DAP10), white-pink fruits (F-DAP18), pink fruits (F-DAP28), and red ripe fruits (F-DAP34) (Figure 5B). The transcription levels of 10 *ClBBXs* (40%) were relatively low at different stages. For instance, *ClBBX28* was almost not expressed in fruits, while *ClBBX10*, *16* and *21c* were not detected in F-DAP28, and their levels were also very low at the other three fruit stages. Differently from the stable low expression pattern of *ClBBX6b* in different stages, *ClBBX3*, *19b* and *30a* all had the highest levels in F-DAP18. The levels of the other 15 members in fruits were high. Among them, seven genes (*ClBBX5*, *6a*, *7b*, *12*, *19a*, *22*, and *29a*) maintained high levels at different stages. The transcription levels of *ClBBX7a*, *14* and *20* were higher in early stages (F-DAP10 and F-DAP18) than in late periods (F-DAP28 and F-DAP34), whereas the reverse was true for *ClBBX15*. The levels of *ClBBX24* and *30b* were lowest in F-DAP10, while those of *ClBBX29b* were the lowest in F-DAP28 (Figure 5B).

### 2.6. ClBBX Expression Profiles in Response to Exogenous SA and MeJA

**Figure 6:**
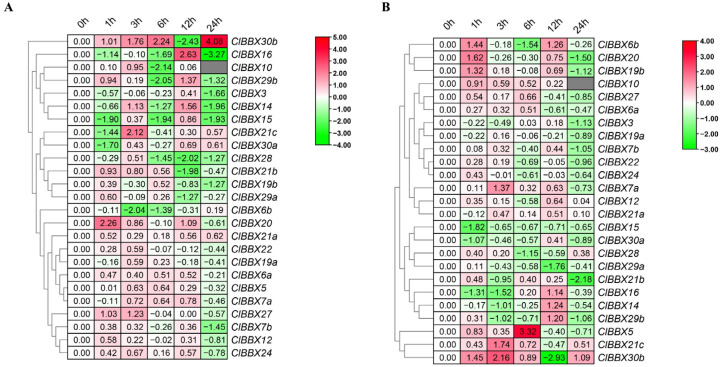
Effects of SA and MeJA on the transcription of *ClBBXs*. The relative expression levels of *ClBBXs* in leaves treated with 100 μM SA and MeJA are shown in (**A**,**B**), respectively. The relative expression levels of *ClBBXs* are compared with those of the untreated plants at corresponding timepoints. The numbers 0, 0.5, 1, 3, 6, 12, and 24 indicate the time (hour) after treatments. The gray blocks indicate that the values were not detected.

The promoters of *ClBBXs* contained many phytohormone response elements. To explore the response of *ClBBXs* to plant hormones, the effects of SA and MeJA treatment on their transcription levels were analyzed (Figure 6). As shown in Figure 6A, 1 h after SA treatment, three (*ClBBX20*, *27*, and *30b*) and four (*ClBBX15*, *16*, *21c*, and *30a*) genes were up-regulated and down-regulated, respectively, while the expression of other genes was not significantly changed (more than two-fold). With the extension of time after treatment, the levels of many genes showed obvious fluctuations. For example, the level of *ClBBX30b* was lowest at 12 h and highest after 24 h, while that of *ClBBX14* and *ClBBX16* was the exact opposite. The levels of *ClBBX10*, *21c* and *27* were the highest after 3 h, while that of *ClBBX6b* was the lowest. Briefly, 6 h after SA treatment, only one (*ClBBX30b*) and seven (*ClBBX6b*, *10*, *14*, *15*, *16*, *28*, and *29b*) genes and were significantly up- and down-regulated, respectively. 

However, the effect of MeJA on the expression of this family genes was relatively weak, which was reflected in the number of *ClBBXs* significantly regulated at different times after MeJA treatment (Figure 6B). Four up-regulated genes (*ClBBX6b*, *19b*, *20*, and *30b*) and three down-regulated genes (*ClBBX15*, *16*, and *30a*) were identified 1 h after MeJA treatment. Briefly, 3 h after treatment, *ClBBX7a*, *21c* and *30b* were up-regulated, and their levels reached the peak, but *ClBBX14* and *16* were down-regulated and showed the lowest levels. At 6 h, *ClBBX6b* and *28* had the lowest levels; however, only *ClBBX5* was up-regulated and peaked, with a nearly 9-fold increase. The levels of *ClBBX14*, *16* and *29b* reached the highest at 12 h, while those of *ClBBX29a* and *30b* reached the lowest. At 24 h, the number of significantly down-regulated genes (7) was the highest and their levels were also the lowest, whereas only *ClBBX30b* was significantly up-regulated.

### 2.7. Expression of ClBBXs under Abiotic Stresses

**Figure 7:**
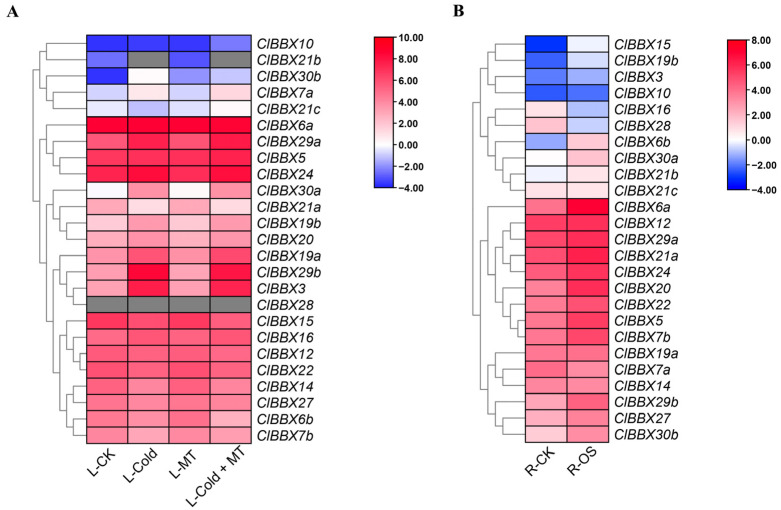
Expression of *ClBBXs* under cold and osmotic stress conditions. Raw data were from FPKM values of watermelon leaves (cultivar Y134) treated with melatonin (150 μM for 3 d) and cold stress (4 °C for 36 h) (**A**), and roots (cultivar M08) treated with 20% PEG6000 for 6 h (**B**). The FPKM values for zero are indicated in gray, and the other data are normalized using log2. L, leaves; R, roots; MT, melatonin; OS, osmotic stress; CK, control tissues.

The RNA-seq data of leaves treated with melatonin (150 μM for 3 d) and cold stress (4 °C for 36 h) were used to analyze the effects of combined melatonin and cold treatment (MT + Cold) on the expression of *ClBBXs* (Figure 7A). As a special case, *ClBBX28* was almost not expressed in leaves under control conditions (L-CK), and it did not respond to melatonin (L-MT) and/or cold stress (L-Cold). Similarly, *ClBBX21b* was not detected in L-Cold and L-Cold + MT tissues. Compared to L-CK, eight and three genes were significantly induced and inhibited by cold stress, respectively. Among the 11 genes, the up-regulated genes included *ClBBX3*, *7a*, *19a*, *19b*, *29a*, *29b*, *30a* and *30b*, while the down-regulated genes were *ClBBX7b*, *14* and *21a*. Except for the induced expression of *ClBBX30b*, almost all *ClBBXs* did not respond to melatonin. In contrast to cold treatment, the combined melatonin and cold treatment only induced the expression of *ClBBX10* and *21c*, and inhibited *ClBBX6b* and *30b*. These data indicate that the role of *ClBBX* family genes in the melatonin-mediated cold stress response pathway may be limited or indirect. Moreover, the RNA-seq data of watermelon roots treated with PEG6000 (20% polyethylene glycol 6000 for 6 h) were used to analyze the effects of osmotic stress on *ClBBX* expression. As shown in Figure 7B, 14 of 25 genes (56%) were drastically induced by osmotic stress, including *ClBBX5*, *6a*, *6b*, *7b*, *15*, *19b*, *20*, *21a*, *22*, *24*, *27*, *29b*, *30a*, and *30b*. Only *ClBBX16* and *28* were inhibited by PEG6000 treatment, and the other nine genes were not sensitive to osmotic stress.

**Figure 8:**
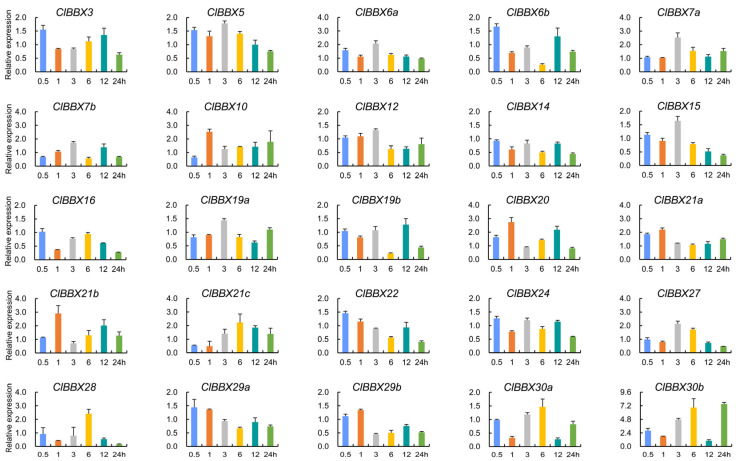
Expression patterns of *ClBBXs* under salt conditions. Seedlings were treated with 100 mM NaCl for 0.5, 1, 3, 6, 12 and 24 h, respectively. Samples without stress treatment at the same time served as the control. Expression data were normalized with *ClYLS8* and *ClACT* as the reference genes.

Many plant BBXs were involved in salt stress [3]. To investigate the expression profiles of *ClBBXs* under salt conditions, the levels of each *ClBBX* in watermelon (XN-8) leaves treated with salt (100 mM NaCl for 24 h) were monitored via qRT-PCR (Figure 8). The levels of seven genes (*ClBBX3*, *5*, *7b*, *12*, *19a*, *24*, and *29a*) did not change significantly within 24 h for salt stress treatment. The levels of seven genes were up-regulated, among which four genes (*ClBBX10*, *20*, *21a*, and *21b*) and two genes (*ClBBX6a* and *7a*) peaked at 1 h and 3 h, respectively, while *ClBBX30b* reached the highest level at 24 h with a 6.5-fold increase. Eight genes were down-regulated under salt conditions, and *ClBBX29b* and *30a* decreased sharply at 3 h and 12 h, respectively. Two (*ClBBX6b* and *19b*) showed the lowest levels at 6 h, while the lowest levels of four other genes genes (*ClBBX14*, *15*, *16*, and *22*) were delayed until 24 h. Additionally, *ClBBX27* peaked at 3 h and decreased significantly at 24 h, and both *ClBBX21c* and *28* decreased significantly at 1 h and peaked at 6 h, but the lowest levels of *ClBBX28* appeared at 24 h. Therefore, *ClBBXs* may play a synergistic role in the watermelon salt stress response.

## 3. Discussion

Plant BBXs play key roles in regulatory networks controlling biological processes. BBX proteins belong to zinc finger TFs, and a thorough understanding of the function of each protein in physiological processes will facilitate their applications in crop genetic improvement [3]. Therefore, the characterization of *BBX* members in different crops is particularly important. With whole genome sequencing, analysis of *BBX* family has been performed in many species. However, identification of watermelon *ClBBX* family is still lacking.

In this study, 25 watermelon *ClBBXs* were systematically identified. The number was same as that in grapevine [8] and pear [24], while it was smaller than that in *Arabidopsis* (32) [7], tomato (29) [22], potato (30) [23], *Ipomoea trifida* (34) [27] and rice (30) [28]. Nonetheless, the number was higher than that in pepper (23) [25], cucumber (22) [29], and *Melilotus albus* (20) [30]. Although the total numbers of BBXs in watermelon, grape and pear were the same, the members in different clades varied greatly (Figure 2; Table S3). For example, the number of ClBBXs was three less than that in grapevine in Clade II, but one more than that in grapevine in Clade I, III and IV. Watermelon and pear had different numbers of BBXs in all five clades except for Clade II. *Arabidopsis* had more BBXs than watermelon did in all clades except for Clade V. Tomato mainly had an advantage in Clade I and II, with two more members in each clade than watermelon. Although the protein number in Clade I, II, III and IV of rice was higher than that in potato, there were five members less than that in potato in Clade V. The remarkable variation in the protein number of the same clades may reflect species-specific duplication or deletions during evolution [8]. 

BBXs contained one or two B-box motifs (B-box1 and B-box2), and sometimes also had a CCT domain [3]. Five Cys residues and two His residues were highly conserved in the two B-box domains of watermelon ClBBXs (Figure 1), which was consistent with those of *Arabidopsis* [7], pear [24], and rice [28]. It was noteworthy that the B-box1 domain of ClBBX6b did not contain the second His residue, and the amino acids between the two His residues were quite different from those of other ClBBXs. The B-box2 domains of ClBBX7a and ClBBX7b also lacked the second His, and the first His in the B-box2 domain of ClBBX10 was replaced by alanine (Ala). A similar case also occurred in the B-box2 domain of grapevine VviBBX9 and VviBBX10, where the first His was replaced by asparagine (Asn) [8]. These variations in conserved domains may confer new functions to BBXs. In addition to the three conserved domains mentioned above, six *Arabidopsis* AtBBXs and five potato StBBXs of Clade I also contained a valine–proline (VP) motif of 6-amino acid (G-I/V-V-P-S/T-F) at the C-terminal, which may be important for protein interaction [3,23]. Among the four watermelon members of Clade I, ClBBX3, ClBBX5 and ClBBX6a had the VP motifs, whereas an atypical motif (G-V-V-P-S-L) was found at the C-terminal of ClBBX6b. Based on the domain structures of BBXs, ClBBX3 with B-box1 and CCT domains was presumptively classified into Clade III; however, it was phylogenetically in Clade I, which contained two B-box domains and a CCT domain (Table 1). This suggests that ClBBX3 lost B-box2 in recent evolutionary events, but retained other common domains of Clade I [6]. Similar exceptions were also found in apple [31] and *Ipomoea trifida* [27]. 

Gene structure was related to the phylogenetic relationship of a gene family [32]. Among the *ClBBXs*, five genes were intronless, nine genes had one intron, four members contained two introns, six members had three introns, and only one gene contained four introns. Genes belonging to Clade I and III had only one intron, genes belonging to Clade 2 had three introns, and genes without introns were mainly distributed in Clade V. The genes in Clade IV had the greatest variation in introns, ranging from zero to three, which is consistent with the expansion and diversification of Clade IV members (Table 1; Figure S1). The percentage of *ClBBXs* without introns (20%) was much higher than that of grapevine (12%) [8], pear (12%) [24], pepper (4.3%) [25], and tomato (6.9%) [22]. *ClBBXs* containing similar gene structures clustered together in phylogenetic trees, such as the genes with no introns (*ClBBX30a* and *30b*), genes with one intron (*ClBBX3* and *5*, *ClBBX6a* and *6b*, *ClBBX14* and *15*) and genes with three introns (*ClBBX7a* and *7b*, *ClBBX19a* and *19b*) (Figure S1), indicating that exon–intron structures support the phylogenetic relationships of the ClBBX family to some extent. Segmental and tandem genomic duplication events contributed to expansions of gene family members [33,34]. In this study, nine segmentally duplicated watermelon *ClBBX* gene pairs were found, but no genes were found in tandem (Figure S2; Table S1), indicating that segmental duplication was the main factor of *ClBBX* family expansion. This is similar to the expansion of *BBX* families in tomato [22] and pear [24]. Twenty-seven orthologous *BBX* gene pairs resulting from segmental duplications were identified between watermelon and *Arabidopsis* (Figure 3; Table S2), which was nearly the number of gene pairs between grapevine and *Arabidopsis* (26) [8], suggesting that they may have evolved from a common ancestor.

To investigate the potential function of watermelon ClBBXs in growth and development, the expression of *ClBBXs* in various tissues was analyzed. The levels of most *ClBBXs* varied greatly in roots, stems, true leaves and cotyledons (Figure 5A), and showed dynamic expression patterns in fruits at different stages (Figure 5B). For example, *ClBBX28* was mainly accumulated in roots, and *ClBBX30b* was preferentially expressed in roots and fruits, while *ClBBX14* and *ClBBX15* were accumulated at higher levels in cotyledons, true leaves and fruits. These data indicated that *ClBBXs* might have functional redundancy in the biological processes of different tissues. *Arabidopsis AtBBX4* positively regulated photomorphogenesis, lateral root development and shoot branching, but negatively regulated flowering [35], suggesting that *AtBBX4* plays important roles in the biological processes in different tissues. *ClBBX5* and *AtBBX4* were a group of orthologous *BBX* gene pairs between watermelon and *Arabidopsis* (Figure 3; Table S2); therefore, *ClBBX5* might have a similar function to *AtBBX4* [8]. In fact, *ClBBX5* accumulated in all tissues of the seedlings and was highly expressed in fruits at different maturity stages (Figure 5 and Figure 7A), implicating that *ClBBX5* was involved in the growth and development of watermelon multi-tissues. Nevertheless, the functions of each *ClBBX* in different tissues need further study.

Plant BBX family members play important roles in hormone signaling pathways [36]. The expression of *BBXs* in many plants was regulated by ABA, SA and MeJA [37,38,39], which were closely related to hormone response elements contained in their promoters. In this study, the response of *ClBBXs* to SA and MeJA was mainly analyzed. Briefly, 14 *ClBBX* promoters contained SA response element as-1 and 11 gene promoters contained a TCA element, accounting for 56% and 44%, respectively. Seven genes, accounting for only 35%, did not contain SA response elements in the promoters. Fourteen *ClBBX* promoters contained MeJA response elements (CGTCA-motif and TGACG-motif), accounting for 56% (Figure 4; Table 2). Uniformly, most genes were responsive to SA and MeJA (Figure 6A). *ClBBX30b* containing one as-1 and one TCA element was significantly down-regulated and up-regulated at 12 h and 24 h after SA treatment, respectively. However, *ClBBX15* containing three as-1 elements and one TCA element was down-regulated at 1 h, 6 h and 24 h after SA treatment. *ClBBX15* and *30b* contained three and one MeJA response elements in promoters, respectively, both of which were regulated by MeJA. It follows that *ClBBXs* may be involved in the interaction of different hormone signals, consistent with that in rice [28] and *Iris germanica* [39]. Although the *ClBBX6b*, *16* and *21b* promoters did not contain the above elements, they were still significantly responsive to SA and MeJA. The possible reason was that cooperation and crosstalk between hormone signaling pathways had overlapping effects on cellular processes [40], thereby indirectly regulating gene expression. 

In addition to growth development and hormonal signaling, BBXs were also involved in abiotic stress responses [3,36]. All *ClBBX* promoters had at least two stress-responsive elements, and MYB (dehydration and the ABA signal-responsive element) and MYC (abiotic stress signals-responsive element) existed in the promoter of each gene, implying their potential functions in the stress response [22]. Four members (*ClBBX19b*, *29b*, *30a*, and *30b*) were markedly regulated by salt, cold and osmotic stress (Figure 7A,B and Figure 8). Their promoters contained many stress response elements; for instance, the promoter of *ClBBX19b* had all the stress response elements listed except STRE, while the *ClBBX29b* promoter contained the largest number of STRE elements (Figure 4). It was noteworthy that *ClBBX30b* was the gene with the highest level induced by salt stress, although it contained a few stress response elements (one STRE, one W box, one MYC and three MYB elements). It was suggested that gene expression may depend on the number, type and location of *cis*-acting elements in promoters. Other *ClBBXs* not regulated by stress may also indirectly participate in the abiotic stress response. *Arabidopsis BBX24/STO* was not induced by salt, but the overexpression of *BBX24/STO* enhanced the salt tolerance of transgenic plants, suggesting that this gene participated in salt stress responses [41]. Therefore, a comprehensive understanding of the function of each ClBBX may be an indispensable link in the analysis of hormone signaling networks and stress signaling pathways in watermelon.

Specially, the levels of *ClBBX21b*, *28* and *30b* in roots were much higher than those in aboveground tissues (Figure 5A), which was consistent with their expression induced by salt stress (Figure 8). Because roots are the first tissues to sense salt stress, their high levels may help improve the response ability of watermelon roots to salt stress. In addition, their different responses to SA, MeJA, cold, and osmotic stress (Figure 6 and Figure 7A,B) suggested that these ClBBX proteins might be involved in stress hormone signaling as transcriptional regulators. Next, it will be an important task to verify the subcellular localization, transcriptional activity, protein interactions and downstream target genes of these key ClBBX TFs (ClBBX19b, 21b, 28, 29b, 30a, and 30b). This will provide more evidence to understand the functional differences of ClBBXs in transcriptional regulation and protein interaction regulation. At the same time, the regulation of the above ClBBXs using overexpression and genome editing technologies may be an important direction to obtain ideal agronomic traits of watermelon.

## 4. Materials and Methods

### 4.1. Sequence Retrieval and Family Member Identification

To identify potential watermelon BBX family members, the hidden Markov model (HMM) profile for the B-box-type zinc finger domain (PF00643) was downloaded from Pfam (https://www.ebi.ac.uk/interpro/entry/pfam, accessed on 12 June 2023) and used as a query to search watermelon (97103) genome v2 in CuGenDB (http://cucurbitgenomics.org/, accessed on 12 June 2023). The amino acid sequences of the *Arabidopsis* BBXs [7] were also used as query sequences to search against CuGenDB. All output putative watermelon BBX proteins with an e-value of ≤0.01 were collected and confirmed using Pfam and SMART (http://smart.embl-heidelberg.de/, accessed on 12 June 2023) [42]. The predicted proteins lacking B-box domains were rejected. The identified genes were assigned as *Citrullus lanatus BBX* (*ClBBX*) genes, and annotated based on their homology with *Arabidopsis AtBBXs*. Sequences of BBXs from *Vitis vinifera* (VviBBXs) [8], *Pyrus bretschneideri* (PbBBXs) [24], *Solanum lycopersicum* (SlBBXs) [22], *Capsicum annuum* (CaBBXs) [25], *Solanum tuberosum* (StBBXs) [23], and *Oryza sativa* (OsBBXs) [28] were obtained from genome databases for each species.

The number of AAs, MW, pI and instability index (with a value of >40 considered unstable) [43] of ClBBXs were analyzed using EXPASY (https://www.expasy.org/, accessed on 12 June 2023). The chromosomal positions and intron numbers of *ClBBXs* were retrieved from CuGenDB. The subcellular locations were predicted via WoLF PSORT (https://wolfpsort.hgc.jp/, accessed on 12 June 2023).

### 4.2. Phylogenetic Analysis and Sequence Alignment

The CLUSTALW program was used to align the full sequences of BBXs. The NJ phylogenetic tree was constructed using MEGA-X [44]. The parameters were as follows: the bootstrap test replicated 1000 times, pair wise deletion, and a Poisson model. The IDs of BBXs from various species are shown in Table S3. The Pfam, SMART, and InterProscan (http://www.ebi.ac.uk/interpro/search/sequence/, accessed on 12 June 2023) programs were used to identify the conserved domains. Weblogo 3 (https://weblogo.threeplusone.com/, accessed on 12 June 2023) was used to create the sequence logos [45].

### 4.3. Chromosomal Location, Gene Structure, and Synteny Analysis

The chromosomal locations of *ClBBXs* were identified based on their chromosomal position and the relative distance derived from CuGenDB. The diagrams of exon–intron structures of watermelon *ClBBXs* was generated using Gene Structure Display Server 2.0 (GSDS, http://gsds.gao-lab.org/, accessed on 12 June 2023) [46]. Synteny analysis and chromosomal location diagrams were generated using TBtools-II v1.120 software [47]. Nonsynonymous (Ka) and synonymous (Ks) rates (Ka/Ks) were calculated using the TBtools software. The Ks value was converted into divergence time in million years based on a rate of 6.56 × 10^−9^ substitutions per site per year. The divergence time (T) was calculated based on the following formula: T = Ks/(2 × 6.56 × 10^−9^) × 10^−6^ million years ago [48].

### 4.4. cis Element Analysis for ClBBX Promoters and Gene Ontology Annotation

The regions (2000 bp upstream of the transcription start site ATG) of *ClBBX* promoters were submitted to PlantCARE online (http://bioinformatics.psb.ugent.be/webtools/plantcare/html/, accessed on 12 June 2023) for *cis* element prediction. With the intention to perform functional annotation identification, 25 ClBBX protein sequences were uploaded to AmiGO 2 (http://amigo.geneontology.org/amigo/landing, accessed on 12 June 2023) and were utilized for gene ontology (GO) analysis. The GO analysis of ClBBXs contains biological process, molecular function, and a cellular component.

### 4.5. Expression Profiles of Watermelon ClBBX Genes Based on RNA-Seq Data

FPKM values for watermelon *ClBBXs* were retrieved from CuGenDB. Specifically, the data for fruits of cultivar Dumara were obtained 10, 18, 28, 34 days after pollination (DAP10, 18, 28, and 34) [49]. The osmotic stress data were obtained from roots of cultivar M08 treated with 20% polyethylene glycol 6000 for 6 h [50]. The leaf data for cultivar Y134 treated with melatonin (150 μM for 3 d) and cold stress (4 °C for 36 h) were also collected [51]. The FPKM values for *ClBBXs* are listed in Table S4. The normalized expression values for each *ClBBX* were used for drawing a heatmap.

### 4.6. Plant Materials and Treatments

Watermelon cultivar XN-8 was used for the expression analysis of *ClBBXs*. Seedlings were grown under the condition of 28 °C (day for 16 h)/22 °C (night for 8 h) for 30 d. The roots, stems, true leaves and cotyledons from seedlings were collected for tissue expression analysis. To study the effect of phytohormones on gene expression, seedlings were treated with 100 μM SA and MeJA for 0, 1, 3, 6, 12, and 24 h, respectively. SA and MeJA were dissolved in 0.004% ethanol, and control seedlings were treated with 0.004% ethanol. To investigate the effects of salinity on *ClBBX* gene expression, seedlings were treated with Hoagland solution containing 100 mM NaCl for 0.5, 1, 3, 6, 12, and 24 h, and control samples were mock-treated with Hoagland solution only. The leaves were sampled from the treated and untreated plants. The control plants were used as the corresponding controls to avoid the effects of the circadian clock on expression analysis [22]. Samples from four seedlings were collected in triplicate from each of the time points for total RNA extraction.

### 4.7. qRT-PCR Analysis

Total RNA was extracted using a total RNA kit (TIANGEN, Beijing, China). RNA quality and concentration were determined using NanoDrop 2000 Spectrophotometer (Thermo Fisher Scientific, Wilmington, DE, USA). First-strand cDNAs were synthesized via the reverse transcription of 1400 ng of total RNA using the One-Step gDNA Removal and cDNA Synthesis Super Mix kits (TransGen, Beijing, China). The reverse-transcribed cDNA (20 μL) was diluted to a final volume of 200 μL and used for quantitative RT-PCR (qRT-PCR) experiments. Primer Premier 5.0 (PREMIER Biosoft International, Palo Alto, CA, USA) was used to design the primer pairs for each *ClBBX* (Table S5). The specificity of primers was assessed utilizing NCBI Primer BLAST (https://www.ncbi.nlm.nih.gov/tools/primer-blast/index.cgi?LINK_LOC=BlastHome, accessed on 12 June 2023).

ChamQ Universal SYBR qPCR Master Mix (Vazyme, Nanjing, China) was used to perform qRT-PCR reactions on qTOWER3 Series-Real-Time Thermal Cyclers (Analytik Jena, Thüringen, Germany). The total reaction system was 20 μL, including 10 μL of SYBR qPCR Master Mix (2×), 0.4 μL of each primer (10 μM), 2 μL of the cDNA samples, and 7.2 μL of ddH_2_O. The reaction system was as follows: 95 °C for 30 s, 40 cycles of 95 °C for 10 s, and 60 °C for 30 s. The fluorescent signal was measured at the end of each cycle, and the melting curve analysis was performed by heating the PCR product from 60 °C to 95 °C. Watermelon *yellow-leaf-specific proein8* (*ClYLS8*) and *β-actin* (*ClACT*) were used as the reference genes [52]. Three technical replicates were performed for each sample [22]. The levels of watermelon *ClBBXs* were calculated using the 2^−△△CT^ method [53], and heat maps were generated using TBtools software [47].

## 5. Conclusions

In this study, 25 watermelon *ClBBXs* were identified. The comprehensive analysis of the *ClBBX* family revealed the conserved domains, classification, subcellular localization, gene structures, phylogenetic relationships, chromosomal distributions, genomic synteny, *cis* elements in promoters, and expression patterns. Most of *ClBBXs* were differentially expressed in tissues (roots, stems, true leaves, cotyledons, and fruit at different stages). The transcription of some *ClBBXs* was regulated by hormones (SA, MeJA, and melatonin) and abiotic stresses (salt, cold, and osmotic stresses). These data indicate that watermelon *ClBBXs* may be involved in growth and development, hormone signaling pathways, and the abiotic stress response. Detecting the subcellular localization and transcriptional activity of key ClBBXs (ClBBX19b, 21b, 28, 29b, 30a, and 30b) regulated by abiotic stress and hormones, obtaining materials with different *ClBBX* expression levels through genetic transformation and genome editing techniques, and clarifying their functions in hormone and stress-induced signaling pathways will be an important task. Our research not only enriches the BBX family of horticultural crops, but also provides a foundation for the functional characterization of watermelon *ClBBX* family genes.

## Figures and Tables

**Table 1:** The information of watermelon *ClBBX* family members.

Gene ID	97103 V2 ID	Position	CDS (bp)	Introns	Protein (AA)	MW (kDa)	pI	Instability Index	Domains			Structure	Localization Predicted
									B-Box1	B-Box2	CTT		
ClBBX3	Cla97C07G143190.1	Chr07: 30638394–30636131 (–)	1071	1	356	40.43	5.20	59.49	20–57		287–328	I	nucl: 8, cysk: 5, plas: 1
ClBBX5	Cla97C08G157330.1	Chr08: 24910533–24909402 (–)	1014	1	337	36.97	5.94	43.77	6–43	49–86	276–317	I	cyto: 8, chlo: 3, mito: 2, plas: 1
ClBBX6a	Cla97C06G122380.1	Chr06: 24733397–24732174 (–)	1128	1	375	40.91	5.79	40.63	23–60	66–103	302–343	I	chlo: 10, cyto: 3, nucl: 1
ClBBX6b	Cla97C11G218170.1	Chr11: 24100942–24099876 (–)	966	1	321	35.57	7.13	48.88	18–46	59–96	246–287	I	chlo: 10, nucl: 2, mito: 1, extr: 1
ClBBX7a	Cla97C02G036700.1	Chr02: 21427678–21424602 (–)	1254	3	417	45.54	5.20	52.09	5–42	48–86	360–401	II	nucl: 7, chlo: 6, cyto: 1
ClBBX7b	Cla97C02G029560.1	Chr02: 2736907–2733278 (–)	1224	3	407	44.43	5.34	57.92	5–42	48–76	350–391	II	nucl: 13, chlo: 1
ClBBX10	Cla97C02G035020.1	Chr02: 10273189–10271324 (–)	1230	3	409	45.16	5.95	53.18	5–42	48–71	353–394	II	nucl: 10, cyto: 2, mito: 1, vacu: 1
ClBBX12	Cla97C07G142260.1	Chr07: 29814600–29817362 (+)	1479	3	492	54.76	5.73	41.88	14–51	57–94	444–485	II	nucl: 10, cyto: 2, chlo: 1, vacu: 1
ClBBX14	Cla97C05G104580.1	Chr05: 32375147–32373841 (–)	1203	1	400	44.88	5.56	46.50	20–57		345–386	III	nucl: 11, chlo: 1, mito: 1, cysk: 1
ClBBX15	Cla97C08G148200.1	Chr08: 15793602–15791626 (–)	1062	1	353	40.15	7.49	48.99	19–56		297–338	III	nucl: 6, chlo: 4, mito: 3, cysk: 1
ClBBX16	Cla97C06G124360.1	Chr06: 26546880–26544768 (–)	1101	1	366	42.57	5.17	52.05	21–58		318–359	III	nucl: 6, chlo: 4, mito: 2, plas: 2
ClBBX19a	Cla97C07G135430.1	Chr07: 20838921–20840526 (+)	561	3	186	20.66	6.10	56.65	5–42	56–91		IV	cyto: 8, chlo: 5, nucl: 1
ClBBX19b	Cla97C01G019520.1	Chr01: 32467895–32466880 (–)	510	3	169	18.99	6.31	56.64	5–42	56–91		IV	cyto: 14
ClBBX20	Cla97C02G031640.1	Chr02: 4502490–4501395 (–)	816	2	271	30.00	6.78	44.41	5–42	58–95		IV	nucl: 12, cyto: 2
ClBBX21a	Cla97C08G158070.1	Chr08: 25538552–25539674 (+)	918	2	305	32.77	6.00	49.00	5–42	58–95		IV	nucl: 7, chlo: 3, cyto: 3, plas: 1
ClBBX21b	Cla97C11G210750.1	Chr11: 4105063–4104488 (–)	576	0	191	21.30	5.79	69.75	5–42	61–98		IV	nucl: 8, cyto: 4, extr: 1, cysk: 1
ClBBX21c	Cla97C09G176180.1	Chr09: 19493336–19493994 (+)	561	1	186	20.54	5.79	49.30	5–42	62–99		IV	nucl: 10, cyto: 3, extr: 1
ClBBX22	Cla97C07G136800.1	Chr07: 24268936–24266735 (–)	897	2	298	32.55	5.20	55.05	5–42	57–94		IV	nucl: 14
ClBBX24	Cla97C06G110200.1	Chr06: 824640–822408 (–)	714	2	237	26.02	4.95	74.98	5–42	57–94		IV	nucl: 11, chlo: 1, cyto: 1, cysk: 1
ClBBX27	Cla97C05G104170.1	Chr05: 32126998–32124171 (–)	1503	4	500	56.18	6.32	67.06	5–42			V	nucl: 13, cyto: 1
ClBBX28	Cla97C03G056720.1	Chr03: 5408237–5408656 (+)	420	0	139	15.44	5.59	46.17	24–60			V	nucl: 6, chlo: 3, cyto: 3, plas: 1, extr: 1
ClBBX29a	Cla97C05G096260.1	Chr05: 24895894–24897102 (+)	837	1	278	30.41	4.38	67.64	4–40			V	chlo: 6, nucl: 6, mito: 1, plas: 1
ClBBX29b	Cla97C01G022250.1	Chr01: 34046068–34046445 (+)	378	0	125	13.42	4.98	35.69	5–41			V	cyto: 9, chlo: 4, nucl: 1
ClBBX30a	Cla97C02G035010.1	Chr02: 10269554–10269940 (+)	387	0	128	14.02	8.20	28.24	34–70			V	cyto: 6, chlo: 4, mito: 3, nucl: 1
ClBBX30b	Cla97C02G028150.1	Chr02: 1650134–1650535 (+)	402	0	133	14.81	5.76	36.96	33–69			V	chlo: 12, mito: 1, extr: 1

Note: (+) indicates forward strand; (–) indicates reverse strand. Abbreviations: CDS, coding sequence; AA, amino acid; Chr, chromosome; MW, molecular weight; pI, isoelectric point; CTT, CONSTANS, CO-like and TOC1 domain.

**Table 2:** The *cis* elements in the promoters of more than 10 *ClBBXs*.

*cis* Elements	Number of Genes	Functions of *cis* Elements	Type of *cis* Elements
CAAT-box	25	Common *cis*-acting element in promoter and enhancer regions	
TATA-box	25	Core promoter element around -30 of transcription start	
G-Box	23	*cis*-acting regulatory element involved in light responsiveness	Light-responsive
Box 4	25	Part of a conserved DNA module involved in light responsiveness	Light-responsive
GATA-motif	12	Part of a light-responsive element	Light-responsive
GT1-motif	22	Light-responsive element	Light-responsive
AE-box	13	Part of a module for light response	Light-responsive
TCT-motif	14	Part of a light-responsive element	Light-responsive
MRE	12	MYB binding site involved in light responsiveness	Light-responsive
STRE	19	Stress-responsive elements	Stress-responsive
ARE	24	*cis*-acting regulatory element essential for the anaerobic induction	Stress-responsive
W box	12	Wounding and pathogen responsiveness	Stress-responsive
MYB	25	Responds to dehydration and ABA signals	Stress- and hormone-responsive
MYC	25	Responds to abiotic stress signals	Stress-responsive
WRE3	11	Wound-responsive element	Stress-responsive
TC-rich repeats	12	*cis*-acting element involved in defense and stress responsiveness	Stress-responsive
CGTCA-motif	14	*cis*-acting regulatory element involved in MeJA responsiveness	Hormone-responsive
AAGAA-motif	18	*cis*-acting element involved in abscisic acid responsiveness	Hormone-responsive
ABRE3a	16	*cis*-acting element involved in abscisic acid responsiveness	Hormone-responsive
ABRE	22	*cis*-acting element involved in abscisic acid responsiveness	Hormone-responsive
ERE	17	Ethylene-responsive element	Hormone-responsive
TGACG-motif	14	*cis*-acting regulatory element involved in MeJA responsiveness	Hormone-responsive
as-1	14	*cis*-regulatory element induced by salicylic acid	Hormone-responsive
TCA-element	11	*cis*-acting element involved in salicylic acid responsiveness	Hormone-responsive

## Data Availability

Not applicable.

## Supplementary Materials

The following supporting information can be downloaded at https://www.mdpi.com/article/10.3390/plants12142634/s1. Figure S1: Characterization of watermelon *ClBBX* genes; Figure S2: Distribution and synteny analysis of *ClBBX* genes on watermelon chromosomes; Figure S3: GO enrichment analysis of *ClBBX* genes; Table S1: Segmental duplications within watermelon *ClBBX* genes and Ka/Ks ratios analysis of segmental duplicate gene pairs; Table S2: Segmental duplications of *BBX* genes between watermelon and *Arabidopsis* and Ka/Ks ratios analysis of segmental duplicate gene pairs; Table S3: The gene IDs of BBX members from *Arabidopsis*, tomato, pepper, potato, grapevine, pear, and rice; Table S4: The FPKM values for *ClBBX* genes; Table S5: Primer sequences used for quantitative real-time PCR analysis.
