# Supplementary material for: Comprehensive Characterization of B-Box Zinc Finger Genes in Citrullus lanatus and Their Response to Hormone and Abiotic Stresses
Source: Plants (Basel). 2023 Jul 13;12(14):2634. doi: 10.3390/plants12142634 (PMC10386417; doi:10.3390/plants12142634)
Supplement: Supplementary file 1 [file plants-12-02634-s001.zip › plants-2476544-supplementary/Supplementary Figures.pdf]

Supplementary Figures

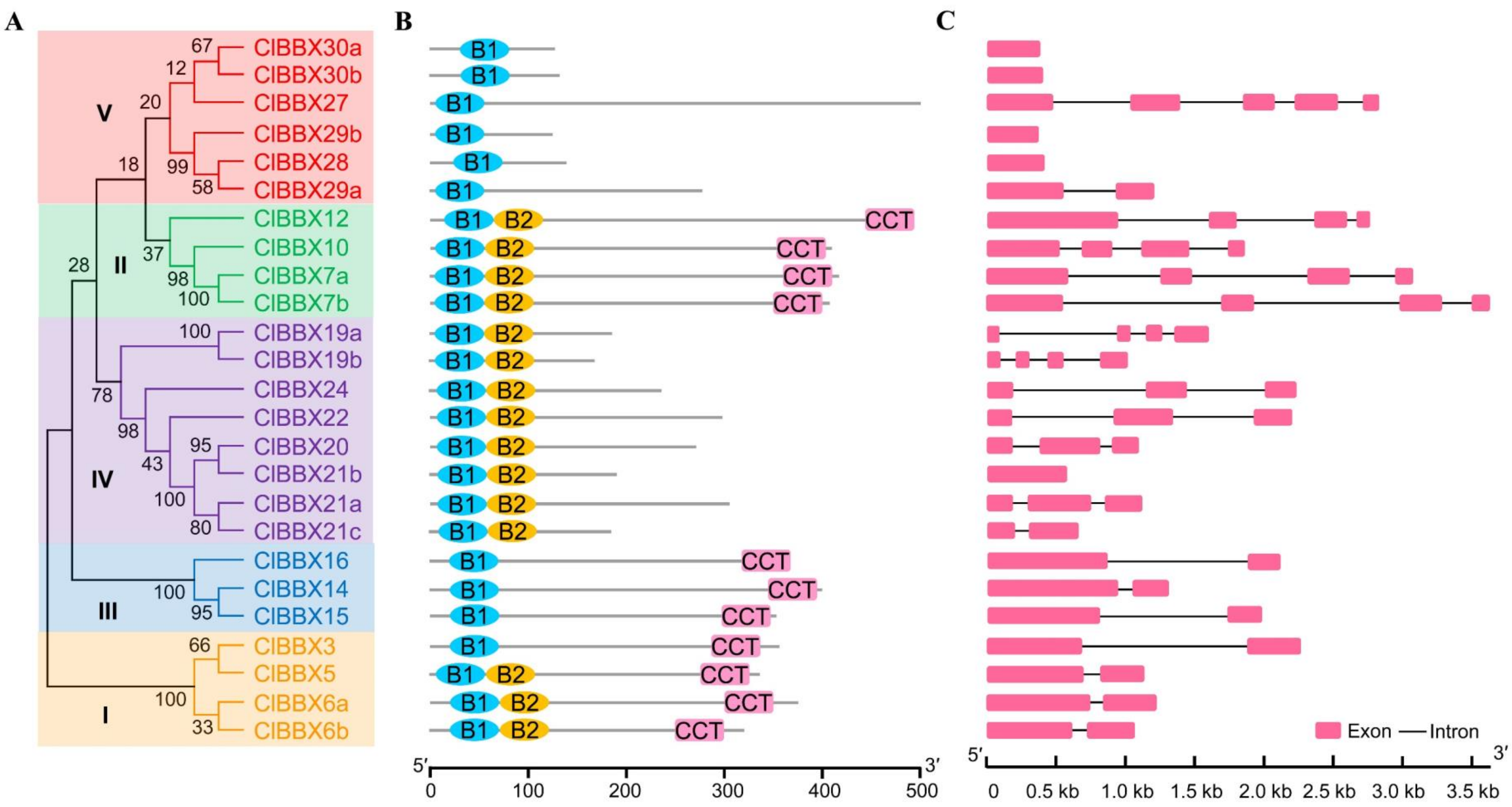

Figure S1. Characterization of watermelon *ClBBX* genes. (**A**) Phylogenetic analysis of ClBBX proteins in watermelon. The five structural groups (I, II, III, IV, and V) were marked by different colors. (**B**) Distribution of conserved domains identified in the 25 ClBBX proteins. (**C**) Exon-intron structure of watermelon *ClBBX* genes. Exons were represented by rose-bengal boxes and black lines connecting two exons represented an intron. The Roman numerals (I-V) indicate the five structural groups

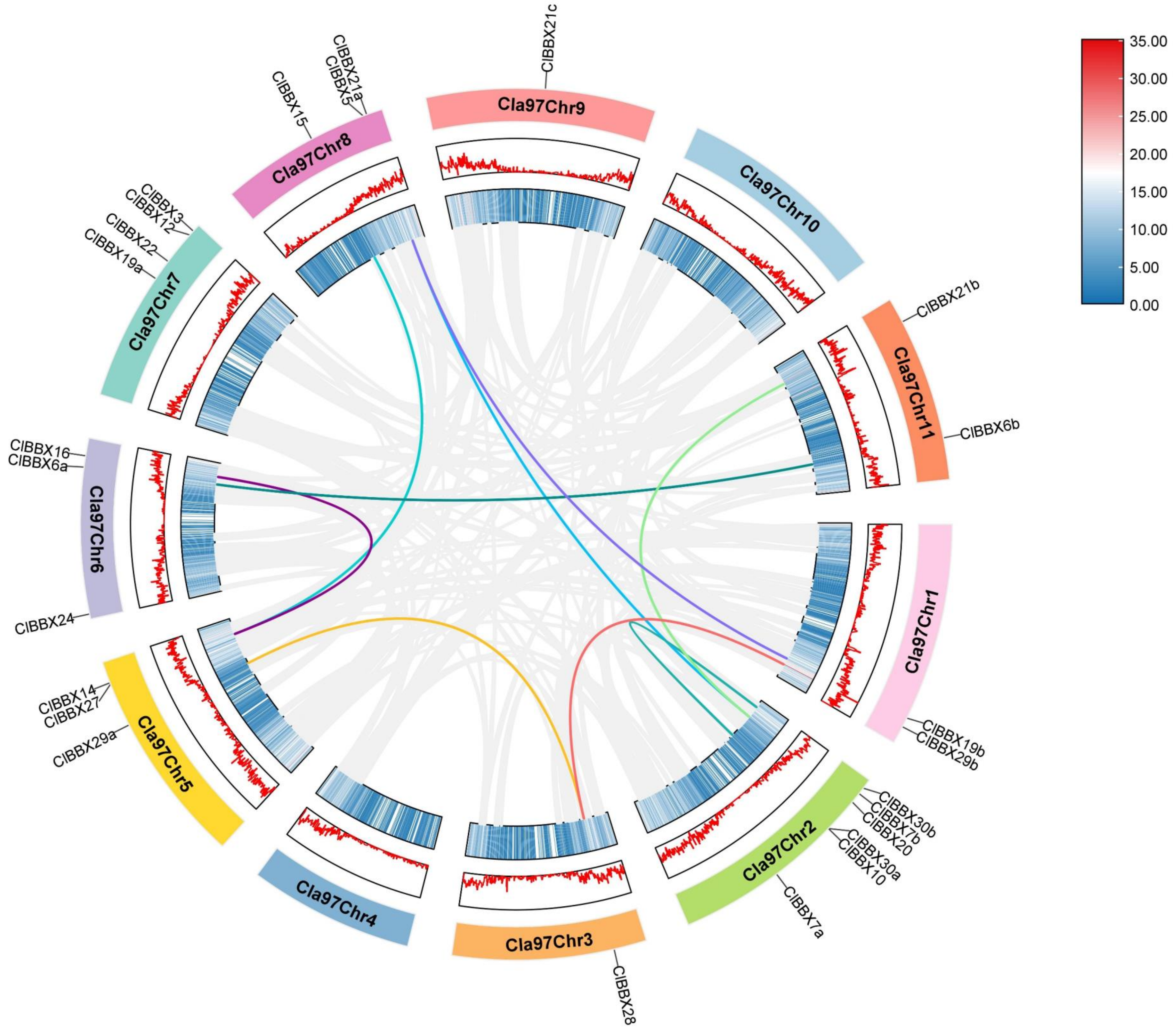

Figure S2. Distribution and synteny analysis of *ClBBX* genes on watermelon chromosomes. The approximate chromosomal locations of the *ClBBX* genes were indicated on the periphery. The colored lines linking genes from different chromosomes denoted segmental duplication events

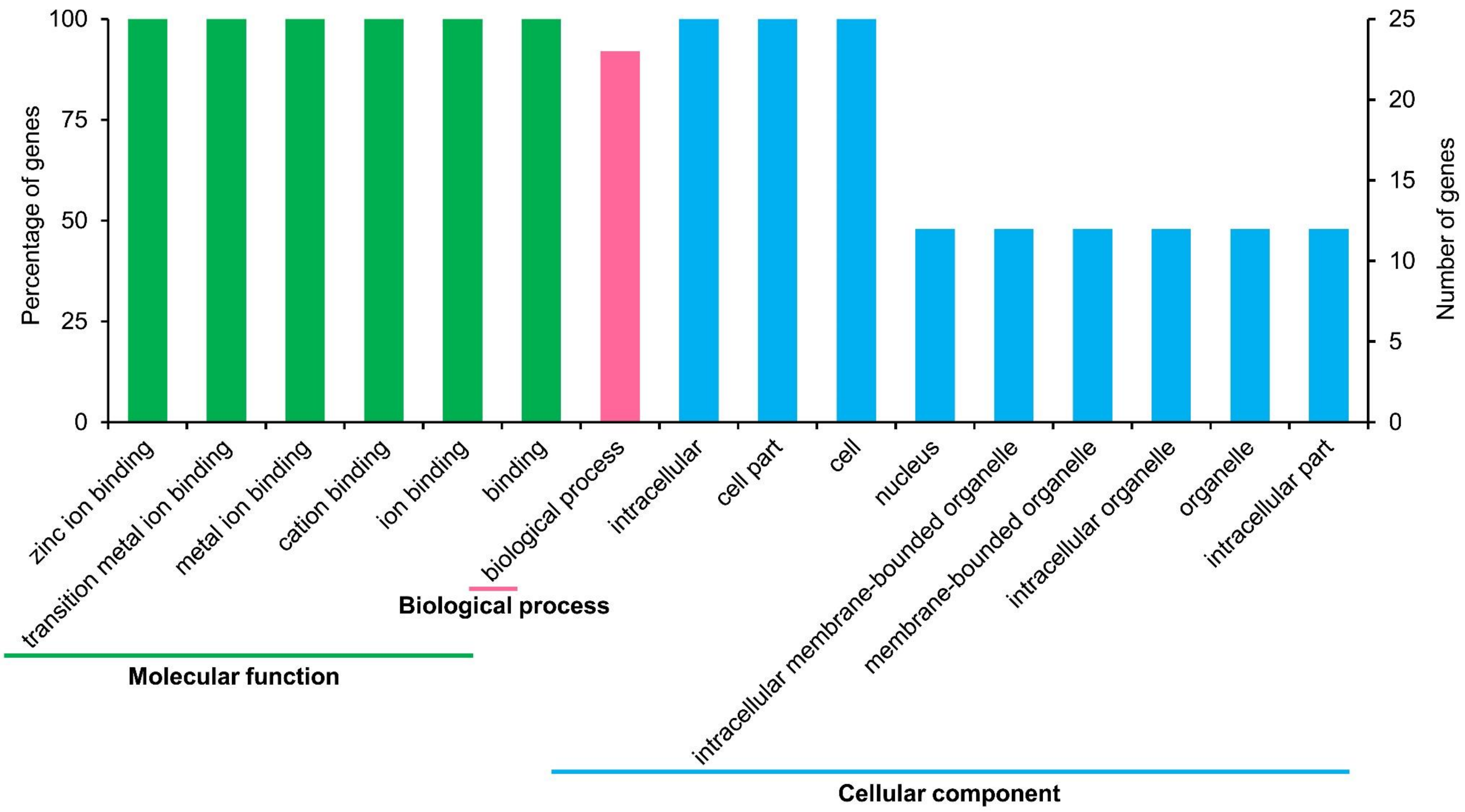

Figure S3. GO enrichment analysis of *ClBBX* genes. The light blue bars represented cellular component, the green bars represented molecular function, and the rose-bengal bars represented biological process.
